# Patient-Controlled Sedation in Port Implantation (PACSPI 1) – A feasibility trial

**DOI:** 10.1016/j.bjao.2022.100026

**Published:** 2022-07-31

**Authors:** Stefanie Seifert, Knut Taxbro, Fredrik Hammarskjöld

**Affiliations:** 1 Department of Anaesthesia and Intensive Care Medicine, Ryhov County Hospital, Jönköping, Sweden; 2 Department of Biomedical and Clinical Sciences, Linköping University, Linköping, Sweden

**Keywords:** feasibility study, patient-controlled sedation, patient satisfaction, propofol, subcutaneous venous port

## Abstract

**Background:**

Central venous access is essential for the administration of chemotherapy and frequent blood sampling in patients with cancer. The subcutaneous venous port is commonly used for this purpose. Subcutaneous venous port implantation is a minor surgical procedure; however, it can provoke pain and anxiety in these vulnerable patients. The aim of this study was, before a full-scale RCT, to determine the feasibility of patient-controlled sedation with propofol and alfentanil as an adjunct to local anaesthesia during SVP implantation.

**Methods:**

We prospectively studied 40 patients scheduled for SVP implantation between 14 April 2021 and 15 October 2021 at a 500-bed secondary level hospital in Sweden. Anaesthesiologists performed subcutaneous venous port implantation with patient-controlled sedation using propofol and alfentanil. We determined pain perception (primary outcome), patient satisfaction, sedation score, and key safety measures.

**Results:**

Of the 40 patients with cancer, 80% reported a pain score ≤3 on an 11-point numeric rating scale during subcutaneous venous port implantation. Overall satisfaction with pain management and operating conditions was graded as 10 of 10 on the numeric rating scale. Four patients (10%) had bradypnoea (<8 bpm) without oxygen desaturation to ≤90%. Rescue sedation was administered to one patient (2.5%).

**Conclusion:**

Patient-controlled sedation with propofol and alfentanil during subcutaneous venous port implantation is feasible and well accepted. Ultimately the efficacy of patient-controlled sedation with propofol and alfentanil needs to be evaluated in an RCT to provide clinicians with evidence-based guidance for choosing the optimal perioperative strategy for subcutaneous venous port implantation.

**Clinical trial registration:**

NCT 04631393.

In Sweden, cancer is diagnosed in more than 60 000 patients annually. A newly diagnosed cancer imposes psychological and physical challenges to the patients, making this group vulnerable to different aspects of their care. Many cancer patients are eligible for chemotherapy administered through a totally implanted venous access device, commonly referred to as the subcutaneous venous port (SVP). According to the Swedish Perioperative Registry, SVP implantation is one of the most common surgical procedures in Sweden.^1^ However, there is no current guidance as to which procedural analgesic strategy is superior during SVP implantation. Several strategies exist (local anaesthesia only, local anaesthesia in combination with analgosedation or general anaesthesia), and practice is likely to be based on local institutional traditions rather than evidence-based guidance. During SVP implantation using local anaesthesia alone, a quarter of patients experience severe pain and discomfort.^2^

Clinician-controlled sedation involves administration of procedural analgosedation by a trained clinician. However, it carries the risk of oversedation and generates higher costs compared with alternative sedation methods.^3^^,^^4^ Patient-controlled sedation (PCS) is an alternative sedation method to clinician-controlled sedation, enabling patients to self-administer and self-regulate their sedation and analgesia during the procedure. The procedural use of PCS with different sedatives and analgesics is well described and regarded as a safe alternative with a lower incidence of analgesic or sedative rescue interventions compared with clinician-controlled sedation in a number of clinical settings.^5^ Propofol and alfentanil, with their short-acting properties, ensure rapid onset and recovery, making them suitable for outpatient procedures.

In this prospective trial, we aimed to examine the feasibility of PCS with propofol and alfentanil for SVP. The objectives to evaluate feasibility were defined *a priori* and consisted of patient self-reported pain perception scores, overall satisfaction scores, sedation scores, and incidence of adverse events. This trial served as a feasibility study before a planned RCT.

## Methods

### Trial design and participants

This trial was approved by the Swedish Ethical Review Authority on 7 July 2020 (Dnr: 2020–02642). The trial was registered at clinicaltrials.gov (NCT 04631393) before trial commencement. The trial protocol is publicly accessible on researchgate.com. At arrival at the preoperative unit printed information was provided to all eligible patients and written informed consent was obtained from all patients participating in the trial. This trial was conducted according to the standards of Good Clinical Practice (GCP) defined by the International Conference on Harmonization, ethical principles with their origin in the Declaration of Helsinki, and all applicable national and local regulations. The trial adhered to the Consolidated Standards of Reporting Trials guidelines for feasibility trials (CONSORT).

The Patient-Controlled Sedation in Port Implantation 1 (PACSPI 1) trial was a prospective feasibility study of the effects of PCS with propofol and alfentanil as an adjunct to local anaesthesia during SVP implantation. Forty patients were included in the trial with the sample size determined as a convenience sample easily being recruited during a short period. Patients aged ≥18 yr who were scheduled for SVP implantation between 14 April 2021 and 15 October 2021 at the Department of Anaesthesia and Intensive Care at Ryhov County Hospital were screened for inclusion. Inability to operate the PCS apparatus, inability to communicate in Scandinavian languages, the need for general anaesthesia, contraindications to sedation as per anaesthesiologist assessment, non-fasting state, inability to establish peripheral vein access, and pregnancy were exclusion criteria. Patients were screened for eligibility by the nursing staff upon arrival to the preoperative unit. Eligible patients were subsequently informed and consented by a physician from the Department of Anaesthesia and Intensive Care.

Participants were instructed on use of the PCS pump (Syramed μSP6000; Arcomed AG, Kloten, Switzerland) by a nurse anaesthetist. The syringe was loaded with propofol 36 ml (10 mg ml^−1^) and alfentanil 4 ml (0.5 mg ml^−1^). Each time the patient pressed the handheld button, an aliquot of 0.5 ml was injected (propofol 4.5 mg/alfentanil 0.025 mg). The injection time was set to 8 s, restricting self-administration to a maximum of 7 bolus doses per minute corresponding to propofol 31.5 mg and alfentanil 0.175 mg per minute. No lockout period was applied. Participants were able to use the pump as soon as it was connected before sterile swabbing was initiated. Carbocaine–adrenalin (10 mg ml^−1^) diluted with sodium bicarbonate (50 mg ml^−1^) was injected into the operative site. Patient observations before the procedure were recorded and the patients subsequently monitored by a nurse anaesthetist during the procedure. Patients were monitored using electrocardiography for HR, noninvasive BP, oxygen saturation (SpO_2_), and ventilatory frequency at 5-min intervals during the procedure. Bradycardia was defined as HR <40 beats min^−1^, tachycardia as HR >100 beats min^−1^, hypotension as systolic BP <90 mm Hg or a decrease of >30% from baseline, hypoxia as SpO_2_ <90% or a decrease of >5% from baseline, and bradypnoea as ventilatory frequency of <8 bpm. Bradypnoea was treated with verbal stimulation. Oxygen desaturation was treated with increased oxygen flow rate. Supplemental oxygen via a capnograph-fitted nasal cannula was administered to all patients at 2 L min^−1^ during the procedure. The cannula served as a tool for ventilatory frequency but not for quantitative capnography registration. The Observer's Assessment of Alertness/Sedation score (OAA/S)^6^ was used to determine the sedation level during the four procedural steps: (1) sterile swabbing, (2) injection of local anaesthetic, (3) catheter tunnelling, and (4) sterile drape removal. The OAA/S is a 6-point scale ranging from 5 to 0 that involves eliciting a patient response to increasingly intense stimuli. Response readily to name spoken in normal tone (5), lethargic response to name spoken in normal tone (4), response only after name loudly/repeatedly called (3), response only after mild prodding (2), response only after painful trapezius squeeze (1), no response after painful trapezius squeeze (0). The operating anaesthesiologist assessed the operating conditions on an 11-point numeric rating scale (NRS) (0=worst possible [i.e. a moving, non-compliant patient putting the patient at risk for complications if procedure is performed] and 10=optimal operating conditions). Puncture attempt was defined as continuous needle advancement to establish vein puncture. Postoperatively, an unvalidated patient perception assessment tool with seven dimensions applying an 11-point NRS ranging from 0 to 10 was used to evaluate patient perception during SVP implantation procedure.

### Outcomes

The primary outcome was maximal pain perception during the procedure as assessed in the recovery unit on NRS before discharge (0=no pain; 10=worst pain imaginable). Secondary outcomes were NRS scores for patient satisfaction with the procedure and pain management (0=not at all satisfied, 10=very satisfied), delivered doses of propofol and alfentanil, time consumption, operating conditions, OAA/S scores during the procedure, and adverse events.

### Statistical analyses

Continuous variables are summarised using descriptive statistics: n (non-missing sample size), median, minimum, and maximum. Frequencies and percentages are reported for categorical variables. Statistical analyses were performed using SPSS version 27 (IBM, Armonk, NY, USA).

## Results

**Fig 1:**
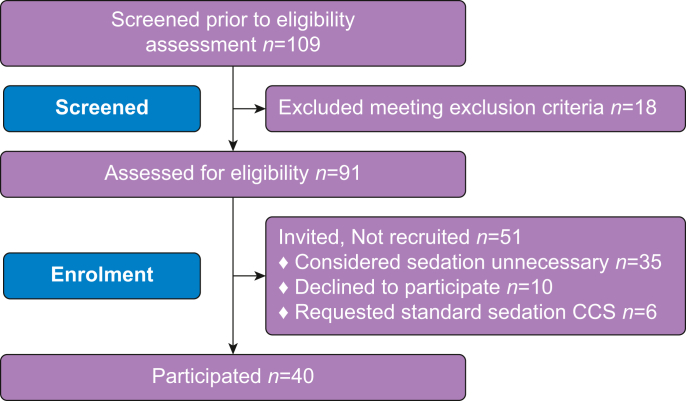
Study outline and flow chart. CCS: clinician-controlled sedation.

In all, 109 participants were screened during the study period. The exclusion criteria applied to 18 patients. Of the 91 eligible patients, 51 (56%) were not recruited. The patient enrolment flowchart is illustrated in Fig 1. Patient baseline characteristics are summarised in Table 1.

**Table 1:** Patient characteristics.

Patient characteristics	*n* (%) or median (min–max)
Age (yr)	66 (37–80)
Sex	
Female	27 (67.5)
Male	13 (32.5)
BMI (kg m^−2^)	25 (19–34)
ASA class	
1	3 (7.5)
2	24 (60)
3	13 (32.5)
Treatment goal for cancer diagnosis	
Adjuvant	26 (65)
Palliative	14 (35)
Previous long-term venous access	5 (12.5)

The preferred vessel for puncture was the right internal jugular vein (87.5%). Arterial puncture and haematoma each occurred in one patient (Table 2). Ultrasound guidance was used for all procedures. The anaesthesiologists' satisfaction with the operating conditions was graded as 10. The procedure characteristics are listed in Table 2.

**Table 2:** Procedure characteristics and complications. LA, local anaesthetic; NRS, numeric rating scale; RA, right atrium; SVC, superior vena cava.

Procedure characteristics and complications	*n* (%) or median (min–max)
Waiting time in preoperative unit (min)	41 (10–215)
Vein choice	
Internal jugular	40 (100)
Laterality	
Right	35 (87.5)
Left	5 (12.5
Ultrasound guidance	40 (100)
LA volume (ml)	30 (10–40)
Number of punctures	1 (1–6)
Arterial puncture	1 (2.5)
Haematoma	1 (2.5)
Pneumothorax	0
Assistance from colleague	1 (2.5)
Procedure aborted	0
Catheter tip position	
Lower 1/3 SVC	31 (77.5)
RA	9 (22.5)
Procedural time (min)	32 (17–84)
Operating conditions (0–10)	10 (5–10)

The median administered doses of propofol and alfentanil were 65.7 (0–243) mg and 0.37 (0–1.36) mg, respectively. The median sedation score was 5 at all procedural time points. Bradypnoea occurred in four patients (10%) and was resolved by verbal stimuli. No hypoxic events were observed. One patient (2.5%) required rescue sedation. Sedation characteristics and adverse events are presented in Table 3.

**Table 3:** Sedation characteristics and adverse events. OAA/S, Observer's Assessment of Alertness/Sedation score; T1, sterile swabbing; T2, injection of LA; T3, catheter tunnelling; T4, sterile drape removal.

Sedation characteristics and adverse events	*n* (%) or median (min–max)
OAA/S score at procedural stage	
T1	5 (3–5)
T2	5 (4–5)
T3	5 (3–5)
T4	5 (1–5)
Delivered volume propofol/alfentanil (ml)	7.3 (0–27)
Delivered propofol (mg)	65.7 (0–243)
Delivered alfentanil (mg)	0.37 (0–1.36)
Rescue sedation	1 (2.5)
Hypoxia (SpO_2_ <90%)	0
Bradypnoea (<8 bpm)	4 (10)
Chin lift	0
Mask ventilation	0
Tachycardia (>100 beats min^−1^)	0
Bradycardia (<40 beats min^−1^)	0
Hypotension (systolic pressure <90 mm Hg)	0
Time in recovery (min)	45 (15–103)

Thirty-two patients (80%) experienced pain levels of 3 or less on the NRS, with a median pain perception of 1 (0–6). Median satisfaction with pain management was 10 (6–10). The patient response regarding the importance of being in control of sedation and analgesia was 8.5 (0–10). The patients' pain perceptions and satisfaction are presented in Table 4.

**Table 4:** Pain perception or patient satisfaction. Data are presented as number (%) and median (minimum–maximum). NRS, numeric rating scale (0–10); Pain (0=no pain, 10=worst pain imaginable); Satisfaction (0=not at all, 10=very).

Pain perception or patient satisfaction	*n* (%) or median (min–max)
NRS score for pain perception ≤3 (0–10)	32 (80)
Maximal pain perception during procedure (0–10)	1 (0–6)
Maximal pain perception in arm with infusion (0–10)	1 (0–8)
Overall satisfaction with pain management (0–10)	10 (6–10)
Overall satisfaction with care (0–10)	10 (9–10)
Satisfaction with staff contact (0–10)	10 (9–10)
How important is sedation? (0–10)	9.5 (0–10)
How important is it to be in control of sedation? (0–10)	8.5 (0–10)

## Discussion

The main finding of this feasibility trial was that PCS with propofol and alfentanil for SVP implantation resulted in low self-reported pain perception and high patient satisfaction and provided good operating conditions during the procedure. To the best of our knowledge, no prior study has examined PCS with propofol and alfentanil in the context of SVP implantation.

Safety for procedural sedation is crucial and has been addressed in several guidelines.^7^^,^^8^ The concept of PCS was first described in 1989.^9^ Since then, the use of PCS has been described in several clinical settings and with different sedative and analgesic regimens.^5^ PCS with propofol is regarded as a safe technique that reduces the risk of oversedation compared with clinician-controlled sedation.^4^ PCS with propofol and alfentanil has been used for procedural sedation in gynaecological and endoscopic settings.^10^^,^^11^ However, data regarding respiratory safety are contradictory. PCS with a combination of propofol and alfentanil was shown to facilitate completion of gynaecological operative procedures compared with propofol alone; however, respiratory status was compromised. In contrast, it was found to have no adverse respiratory effects in the setting of endoscopic retrograde cholangiopancreatography.^10^^,^^11^ In the present trial, alfentanil at 0.025 mg dose^−1^ was associated with limited episodes of bradypnoea in four patients, without oxygen desaturation or the need for mechanical ventilation, suggesting that it might have minor effects on ventilatory frequency. No adverse events related to HR and BP were registered.

Opioids play a crucial role in the relief of procedural pain. Preprocedural buccal administration of fentanyl and short-acting intravenous remifentanil has been shown to decrease patients' pain perception during SVP implantation.^12^^,^^13^ In the present trial, the overall perception of pain was low, and 80% of the patients experienced pain of 3 or less on the 11-point NRS during SVP implantation. Furthermore, the satisfaction with pain management was high. This trial suggests a somewhat lower degree of pain perception in SVP implantation with PCS than with local anaesthesia only.^14^

Interestingly, self-administered doses varied substantially, ranging from 0 to 27 ml, suggesting large inter-individual differences in sedative and analgesic requirements. This is consistent with previous findings in which PCS with midazolam and fentanyl were used for tunnelled venous access.^15^ More than a third of eligible patients declined participation, as they considered sedation for a presumably minor procedure unnecessary or wanted to drive their vehicles home after discharge. This not only generated selection bias, but might reflect patients' varying needs for sedation and analgesia, as mentioned above. The challenge lies in identifying patients according to their sedative needs before the procedure begins. As suggested earlier, the likely determinants for self-administered anxiolytics seem to be the patient's own assessment of their needs in a shared decision-making process agreeing on an individualised analgesic strategy.^16^

Patient satisfaction with care and staff contact was very high. Patient satisfaction is challenging to quantify, and patient-related experience measures are key to providing good patient-centred clinical care and allowing comparison of clinical routines.^17^ Unfortunately, no appropriate validated questionnaire for sedation in minor procedures (such as central venous access) exists, leading to the pragmatic approach of using established methods, although not validated, to facilitate comparison.^15^

The present trial serves as a feasibility study before an RCT and therefore has limitations that must be mentioned. With a small sample size determined as a convenience sample, adverse events were likely missed. However, the safety profile in this trial is similar to other PCS trials suggesting the concept would be usable in a future study.^10^ The lack of a validated patient-related experience measure for central venous access insertion limits proper tools for measuring patient satisfaction and comparison with other studies; this problem needs to be addressed in the future in order to enhance quality in future trials.

## Conclusions

SVP implantation is a common procedure in everyday practice; however, no evidence-based recommendations can currently be made from the existing literature regarding which perioperative analgesic strategy, sedative strategy, or both are superior for patients in need of SVP. This trial suggests that PCS with propofol and alfentanil during SVP implantation is feasible and well accepted. Its efficacy and safety are now to be evaluated in an RCT to provide evidence-based guidance for choosing the optimal perioperative strategy for SVP implantation.

## Funding

Futurum Academy of Health and care, Jönköping County, Sweden (FUTURUM-937005).

## Data availability

The datasets generated during the current study are available from the corresponding author on reasonable request.

## Authors' contributions

Study conception and design: SS, KT, FH.

Material preparation: SS.

Data collection: SS.

Data analysis: SS, KT, FH.

## Declaration of interest

The authors declare that they have no financial or non-financial interests to declare.
